# Do Targeted Interventions Diminish Victimization? Testing the Short- and Longer-term Effectiveness of Condemning, Empathy-Raising, and Combined Approaches

**DOI:** 10.1007/s10964-025-02173-0

**Published:** 2025-04-15

**Authors:** Lydia Laninga-Wijnen, Mark Huisman, Daniel Graf, Christina Salmivalli

**Affiliations:** 1 INVEST/Psychology, University of Turku, Turku, Finland; 2 Statistics, University of Groningen, Groningen, The Netherlands

**Keywords:** Targeted interventions, Bullying victimization, Teachers, Condemning, Empathy

## Abstract

Given the detrimental effects of school bullying, it is essential that teachers are provided with effective guidelines on how to implement targeted interventions if a case of bullying comes to their attention. Yet to date, most research has focused on how bullying can be prevented, rather than how it should be intervened upon. To address this gap, the current study compared the short- and longer-term development of victimization of students whose bullies were enrolled in a targeted intervention, and compared three approaches taken in such interventions when talking to perpetrators: (1) promoting empathy for victims among bullies, (2) explicitly condemning bullying behaviors, and (3) a combination of these approaches. The sample consisted of *n* = 274 victims from primary and secondary schools (56.6% female, Mage = 11.95, SD = 1.89). School personnel used a mobile application *KiVappi to* document the steps they took when implementing targeted interventions on bullying perpetrators of these victims (including a follow up meeting in which victims were asked about the short-term effectiveness of the intervention). Most cases were handled with an empathy-raising approach (*n* = 117), followed by the condemning (*n* = 113) and combined (*n* = 44) approach. Targeted intervention data was matched to survey data collected to examine longer-term changes in self-reported victimization. The results indicate that the success rates of targeted interventions on the short-term were promising: 88.2% of the victims indicated that the victimization had decreased or ceased, and the combined approach seemed to be the “best bet”. In the longer term, victims whose bullies were enrolled in a targeted intervention were equally stable in self-reported victimization as the control group - irrespective of the approach taken in the targeted intervention. Thus, despite potential short-term successes, targeted interventions may not be enough to help victims of bullying escape their plight in the longer term.

## Introduction

Bullying is a persistent problem in many schools, with devastating consequences for all youth involved (Choi & Park, 2021); it is therefore essential that teachers are provided with evidence-based guidelines regarding the best ways to handle bullying cases. Two possible lines of action when discussing the situation with the bullying perpetrators have been introduced in the literature (Garandeau et al., 2014). A *condemning approach* includes holding the bully responsible for the harm caused, and indicating that the behavior is not tolerated by adults at school (Olweus, 1996). An *empathy-raising approach* aims at raising the bully’s empathy for the victimized peer and establishing a shared understanding that the situation needs to be solved (Robinson and Maines, 2008). To date, a few studies compared the relative effectiveness of these two targeted intervention approaches in diminishing victimization or enhancing bullies’ willingness to change their behavior, and found them to be equally effective (Garandeau et al., 2014, 2016; Johander et al., 2021, 2022), with some indication that a combination of condemning *and* empathy-raising (i.e., a *combined* approach) elements might be the “best bet” (Johander et al., 2022). Yet, the measures that previous work used to assess the effectiveness of interventions may be subject to memory bias or lack of ecological validity, and recent work advocates the need of applying a real-life design (with ecological momentary assessments) to reliably test intervention effectiveness (Salmivalli et al., 2025). Moreover, most previous studies focused on the short-term effectiveness (weeks) rather than the long-term effectiveness (across a school year), and did not include a control group in which no targeted intervention was implemented. Therefore, this study applies ecological momentary assessments to examine the relative effectiveness of a condemning, an empathy-raising, and a combined approach in diminishing victimization, both on the short- and longer-term, while including a control group.

### Do Targeted Intervention Approaches Diminish Victimization in the Short- and Long-Term?

Interventions aimed at reducing bullying can be universal or targeted. Universal interventions consist of prevention efforts involving all children, regardless of whether they have been directly involved in bullying. Targeted interventions address children involved as victims or perpetrators in cases of systematic bullying that come to the attention of school personnel. Many school-based programs - such as the KiVa anti-bullying program - involve both universal elements (e.g., student lessons; playground supervision) *and* guidelines for targeted intervention, delineating the approaches teachers can take when discussing the situation with the bullying perpetrators. A *condemning approach* (also referred to as a confronting approach; Garandeau et al., 2014) consists of telling the perpetrator that the adults at school know about their bullying behavior and indicating that the behavior is not tolerated and should stop immediately. This approach thus emphasizes the importance of setting clear and firm limits for bullying, aspects that were already advocated long ago by Olweus in his Bullying Prevention Program (1996). Instead, an empathy-raising approach (also referred to as a non-confronting approach; Garandeau et al., 2014) aims at arousing the bullies’ empathy for victims and establishing a shared understanding that the situation needs to be solved because it is painful for the victim. Bullies are neither blamed nor held responsible for the victims’ harm. This approach is originally derived from the Method of Shared Concern (Pikas, 1989) and the Support Group Method (Robinson and Maines, 2008).

Most evaluation trials have tested the effectiveness of anti-bullying interventions as a whole, rather than separating their universal and targeted components (see Hensums et al., 2023). Meta-analyses of such general evaluation trials suggest that interventions encouraging “punitive disciplinary strategies” (confronting the bullies and insisting that they need to stop) yield significant decreases in bullying behaviors (Ttofi & Farrington, 2011). Instead, interventions encouraging “non-punitive disciplinary strategies” such as raising empathy had an iatrogenic effect on bullying perpetration and victimization (Gaffney et al., 2021). Yet, it should be recognized that the same intervention may have varying instructions for targeted interventions. For instance, in the KiVa program, guidelines for targeted interventions include both condemning and empathy-raising approaches and the schools’ intervention teams are supposed to choose the one they prefer to use. Moreover, teachers often deviate from such guidelines and use their own invented strategies (Johander et al., 2021). Lastly, the meta-analysis does not differentiate to which extent the strategies are used for single events of aggression, or for handling systematic cases of bullying. Bullying is by definition systematic and repeated, and it should be treated – and intervened on – as such: rather than responses to aggressive incidents one-by-one as they happen, targeted interventions should be implemented (see also Salmivalli et al., 2025). Therefore, to better understand the effectiveness of condemning and empathy-raising approaches, they should be compared systematically while also ensuring that adults doing the intervention follow up the guidelines.

To date, only a handful of studies have evaluated the effectiveness of targeted interventions specifically. Two studies examined the short-time effectiveness of targeted interventions in the context of a randomized controlled trial on 1st to 9th grade students, whose schools were enrolled in the Finnish *KiVa intervention* (Garandeau et al., 2014, 2016). Half of the intervention schools were trained to use the condemning approach, whereas the other half was trained to use the empathy-raising approach. In the first study, about two weeks after bullies had been targeted by an intervention, their victims were asked by the teacher whether the bullying had stopped, which was the case in about 78% of the situations. In 98% of the cases, bullying had either decreased or stopped (Garandeau et al., 2014). Both approaches were considered equally effective in stopping victimization, although some factors moderated these effects: the condemning approach worked better than the empathy-raising approach in secondary schools (the two approaches were equally effective in primary schools) and in cases of short-term victimization (i.e., when the victimization had lasted less than 6 months).

A follow-up study tested bullies’ willingness to change their behavior right after an intervention meeting with a teacher (Garandeau et al., 2016). Bullies reported high willingness to change their bullying behavior after these meetings, and this was positively - and equally strongly - affected by the extent to which bullies perceived the teacher as (1) condemning of the bullying behavior (2) attempting to raise their empathy. Notably, bullies’ intention to change their behavior was highest when they felt that their teacher had *both* condemned the bullying *and* aroused their empathy, suggesting that a combination of approaches may be most effective. This finding was replicated in an experimental study on Finnish 7th grade students (Johander et al., 2022), in which students were asked to imagine they had bullied a peer and were invited to a discussion with a teacher. They subsequently were presented a video vignette with either a condemning, empathy-raising, or combined (including both condemning and empathy-raising) message from a teacher. Students’ intention to stop bullying was highest among those who saw the combined message.

Whereas previously mentioned studies examined the short-term effectiveness of targeted interventions, one large-scale study on Finnish schools implementing the KiVa program examined the longer-term effectiveness of a condemning versus an empathy-raising approach, using annual surveys (Johander et al., 2021). Across six years, at the end of each school year, students whose victimization had been intervened on were asked about their perceptions regarding the effectiveness of these interventions (with the question: “Did the adult intervention affect your situation?”). In general, targeted interventions effectively reduced victimization by the end of the school year: in 74% of the cases, the victimization had decreased or stopped according to the victims. Effects were similar for schools who typically used the condemning approach and schools who typically used the empathy-raising approach.

Another longitudinal study on elementary school students compared the short- (weeks) and longer-term effectiveness (one school year) of a *variant* of the empathy-raising approach in the Dutch KiVa trial, namely the support group approach (Van der Ploeg et al., 2016). When a systematic case of victimization came to the attention of teachers, they were asked to form a peer support group of 6–8 children. The group was supposed to involve the bullies and their assistants, defenders or friends of the victim, and prosocial classmates, with the aim of fostering shared concern for the victim’s situation and encouraging bullies to change their behavior. Initially, victimized children were positive about the support group: 55% reported that bullying had decreased and 29% said it had stopped. However, these beneficial effects did not last until the end of the school year. By then, victims *with* a support group experienced more frequent self-reported victimization than those without one. This indicates the necessity of examining both the short- and longer-term development of victimization after targeted intervention implementation, and to compare the development of victimization across intervention *and* control groups.

Though the few previous targeted intervention studies produced valuable insights, there are at least three important limitations. First, previous work was insufficiently able to test the real-life implementation and effectiveness of these targeted interventions. For example, annual reports detailing the school’s general approach (Johander et al., 2021) may be imprecise, as strategies can vary over time and across team members. Experiments (Johander et al., 2022) may lack ecological validity (i.e., generalizability to the real-life school context). Additionally, comparing schools based on the training they received (e.g., an empathy-raising vs. condemning approach) fails to consider that teachers may forget the training content and deviate from instructions (Garandeau et al., 2014; Johander et al., 2021). Therefore, further research is needed to examine the real-life implementation of interventions and to ensure that teachers follow the assigned approach as intended. Recent work emphasizes the need of using ecological momentary assessments to do this (Salmivalli et al., 2025) - for instance with a mobile phone application that is used by school personnel. Such a mobile application can provide real-time access to targeted intervention guidelines during the various meetings with victims and bullying perpetrators, and can ask teachers to instantly report on agreements made or steps taken. It thus prevents memory bias, increases intervention adherance, and ensures ecologically valid data (Salmivalli et al., 2025).

A second limitation is that most studies evaluated the *short-term effectiveness* of targeted interventions (mostly over a period of weeks). This hinders from formulating firm conclusions regarding the *longer-term development of victimization* (e.g., several months after the intervention). In the only study that compared the longer-term effectiveness of condemning and empathy-raising targeted interventions (Johander et al., 2021), the exact time-span between the targeted intervention and the perceived effectiveness could not be determined. That is, at the end of the school year, victims were asked whether the targeted intervention that had taken place somewhere within that school year was effective in decreasing or stopping the victimization. Questions like this (asking about the effectiveness of an intervention) may even cause social desirability bias, because after all effort put in stopping a bullying situation, there may be a strong wish that the “desired” outcome (stopped victimization) has been obtained, creating potential blind spots for signals of continued victimization (Garandeau et al., 2014). Thus, further research is needed that examines the longer-term development of victimization of students whose cases are handled in a targeted intervention, using measures that may prevent such social desirability bias.

A third limitation is that none of the previous targeted interventions studies included a *control group* in which no targeted intervention took place. Thus, the decrease in victimization or the intention to stop bullying that were detected among students after a targeted intervention could also reflect a natural development that may have taken place even without the intervention.

### Covariates: School Level, Biological Sex, and Duration of Victimization

Adopting a developmental perspective is essential when evaluating the effectiveness of targeted interventions. Bullying is particularly prevalent in childhood, potentially due to underdeveloped self-regulation skills and the overt nature of bullying behaviors, which makes them more easily identifiable by teachers (Cook et al., 2010). In primary school settings, children generally perceive teachers as authority figures who enforce rules and norms that should be respected, and the relatively small school environment facilitates staff collaboration in intervention implementation (De Roo et al., 2020). In contrast, adolescence—often coinciding with the transition to secondary education—is marked by an increasing need for autonomy and a heightened emphasis on peer status (Laninga-Wijnen and Veenstra, 2022). Bullying in this stage is frequently used as a means to gain social standing and tends to be more covert (Yeager et al., 2018). Furthermore, secondary schools are typically larger and more decentralized, making it more challenging for staff to reach a consensus on intervention strategies and their implementation (De Roo et al., 2020). Universal anti-bullying interventions were found to be less effective (or to even have adverse effects) in adolescence, possibly because they do not tap into the developmental needs of adolescents (Yeager et al., 2015) and because implementation is more difficult in larger schools (De Roo et al., 2020). Targeted interventions were also perceived as less effective in secondary schools than in primary schools (Johander et al., 2021, 2022). This could especially be the case for targeted interventions following a condemning approach, as they interfere with adolescents’ needs for autonomy (Yeager et al., 2015). Paradoxically, the only previous targeted intervention study evaluating differences between the *specific approaches* found the condemning approach to be more effective than the empathy-raising approach in secondary school (Garandeau et al., 2014). Consequently, the current study will control for school-level (primary versus secondary school) and explore interaction effects of the various targeted interventions with school level.

Biological sex was included as a covariate in this study. The intervention effects of anti-bullying interventions on victimization have sometimes been found to be larger for boys (Eslea & Smith, 1998), and iatrogenic effects of empathy-raising intervention components on victimization have been found to be stronger for girls than for boys (Hensums et al., 2023). Lastly, there is some indication that the condemning approach is less effective in cases of longer-term victimization than in cases of short-term victimization (Garandeau et al., 2014). Therefore, the duration of victimization was entered as covariate, and potential interaction effects between duration of victimization and type of approaches were explored.

## Current Study

Surprisingly little is known about the effectiveness of targeted interventions taken by adults when they find out about ongoing bullying. The few existing targeted intervention studies applied suboptimal measures, predominantly focused on the short-term effectiveness of interventions, and did not include a control group. Therefore, the first aim of the current study was to apply a novel, real-life implementation design -containing ecological momentary assessments - to test the relative effectiveness of a condemning, empathy-raising, and combined approach in diminishing victimization on the *short-term* (i.e., several weeks later). Although findings of one previous experimental study suggest the *combined approach* to be most effective, no hypotheses were formulated, as this is still an understudied topic. The second aim was to examine whether these various approaches related to decreased victimization in the longer term (i.e., several months later), while comparing this to the longer-term development of victimization of students whose situation *had not been addressed* in a targeted intervention: the control group. It was hypothesized that students whose cases had been addressed in an intervention (intervention group) would be more likely to decrease in self-reported victimization over time than students whose situation had not been intervened in (control group). It was explored whether the effectiveness of targeted interventions in diminishing longer-term self-reported victimization varied as a function of the approach taken in this intervention (condemning, empathy-raising, combined). It was anticipated that targeted interventions would be less effective for girls and for cases whose victimization that already endured for a longer time period. Moreover, this study explored the role of school-level (primary versus secondary education) in intervention effectiveness.

## Methods

### Procedure and Participants

Data stem from the Challenge project, which took place in the academic years 2020–2021 (year 1) and 2021–2022 (year 2). This project aimed at identifying factors that may improve the plight of persistent cases of bullying. The Challenge project was approved by the Ethical Board of the University of Turku and consisted of various subprojects. One is the *Targeted Intervention project* for which ecological momentary assessments were obtained from school personnel, who documented on the steps they took when implementing a targeted intervention. Another subproject was a *Longitudinal Survey* in which students reported on their victimization experiences three times across one academic year (T1 = October, T2 = January, T3 = April) during two consecutive school years. For the current study, targeted intervention data were combined with student survey data to gain insights in both the short- and longer-term development of victimization following targeted interventions.

A total of 23 schools participated in the Challenge project. These schools were located in diverse communities across Finland, with student populations ranging from *n* = 170–1050. The largest schools were combined institutes covering both primary and secondary education, often spread across multiple buildings or locations. All the schools were public schools, like the vast majority (>95%) of basic education schools in Finland. Each school contained students from diverse socioeconomic backgrounds, and there was no strong heterogeneity in this diversity among schools. Students typically go to the school in their neighborhood and, despite some socioeconomic differences between neighborhoods, the level of segregation is low in Finland. The Finnish welfare system aims to guarantee good education to all children, regardless of where they live.

#### Targeted Intervention Data Collection Procedure and Participants

All of the schools enrolled in the Challenge project implemented the KiVa anti-bullying program, which includes both universal elements for bullying prevention *and* procedures for targeted interventions. Each school has its own “intervention team” typically consisting of three teachers or other school personnel who are responsible for both targeted interventions at their school. Whereas usually schools could decide upon the approach to be taken for targeted interventions (condemning or empathy-raising), for the Challenge project they were assigned to one out three conditions, matching to the three approaches (condemning, empathy-raising, and combined). The three approaches were not randomly assigned, but based on what schools used as their regular approach. To ensure that intervention team members were up to date about the procedure for targeted interventions, they received a 2-h training in 2020. This training was held via zoom because of the COVID-19 period. The training was repeated for new team members in 2021. In these trainings, school personnel learned about the definition of bullying (i.e., repeated acts of peer aggression characterized by a power imbalance between perpetrator and victim; Olweus, 1996), and they were instructed to implement targeted interventions on such systematic cases of bullying victimization. They learned how to use the *KiVappi* application, and were instructed on how to report on the steps taken during implementation of the intervention. The content of the trainings was identical for all intervention conditions, apart from the instructions regarding discussions with bullies, which either focused on a condemning, empathy raising, or combined message.

**Fig. 1:**
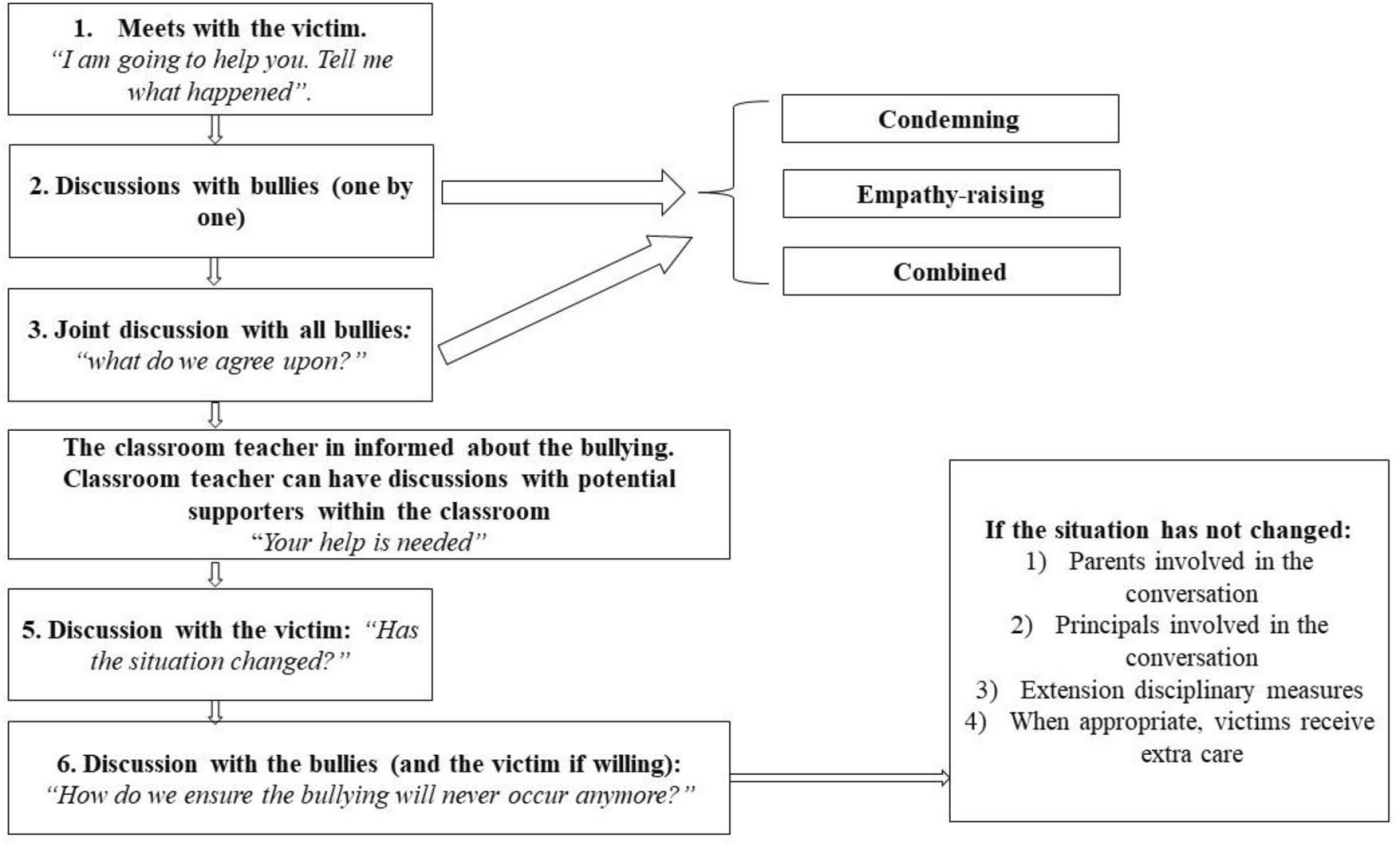
Various steps to be taken during a targeted intervention

**Fig. 2:**
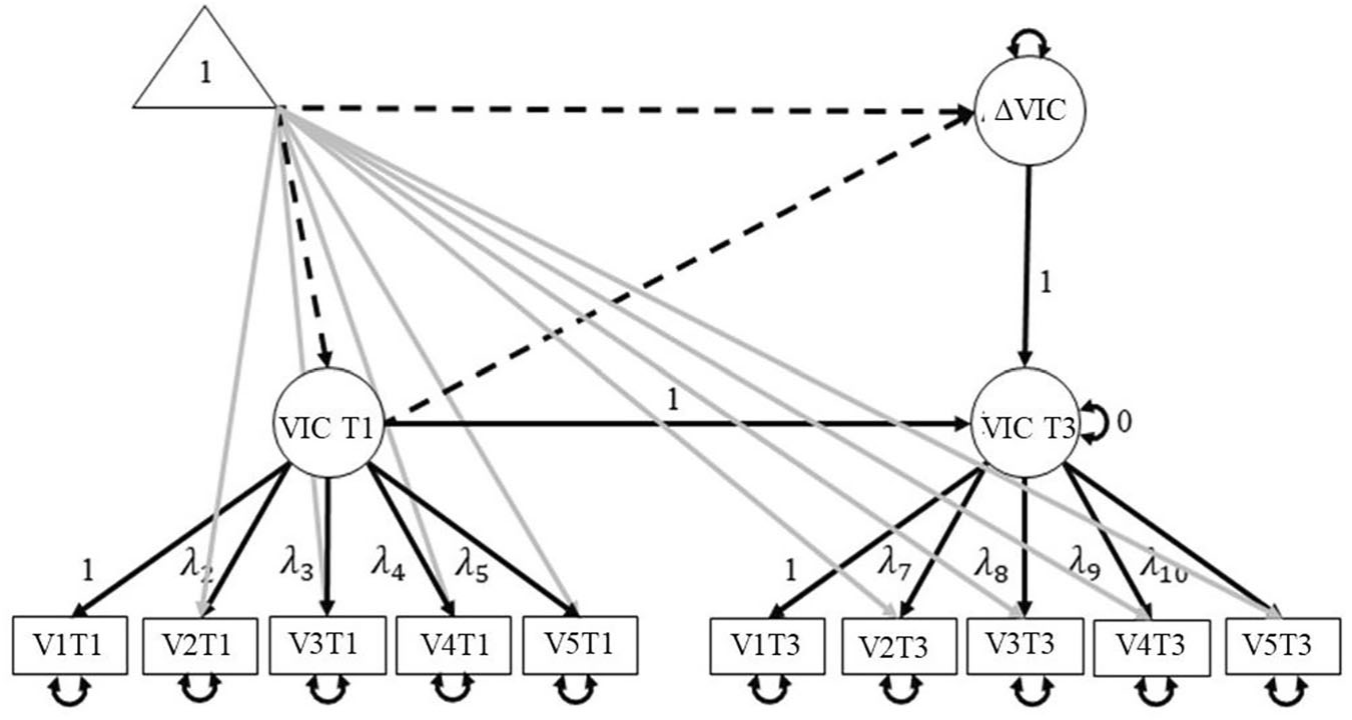
Univariate latent change score model

The intervention team was instructed to take several steps when a suspected case of bullying would come to their attention. As displayed in Fig. 1, the team was supposed to first organize a meeting with the child who had been victimized. Then the team had a meeting with each bully separately, and - in case multiple bullies were involved - a meeting with all bullies together, and during these two meetings the team was instructed to use either a condemning, empathy-raising, or combined approach - depending on the condition their school was assigned to. Furthermore, the classroom teacher was instructed to organize a meeting with a few non-involved, prosocial classmates, to encourage them to support the victim. About two weeks after this intervention, two follow-up meetings were organized: (1) a meeting with the victim separately (meeting 5, Fig. 1), and (2) a meeting with the bullies and (if willing) with the victim together (meeting 6, Fig. 1). During both follow-up meetings, school personnel asked and reported whether the bullying had stopped, decreased, remained the same, or increased, both from the victim’s and the bullies’ perspective. The current study focuses on the *victims’ perspective* on whether the bullying had stopped.

Because one school dropped out from the Challenge project, 22 out of the 23 schools were included in the targeted intervention subproject (*n* = 7 condemning approach, *n* = 8 empathy-raising approach, and *n* = 7 combined approach). Out of these schools, 7 were elementary, 5 were middle, and 10 were combined schools. Across the two academic years, for *n* = 274 victimized students from 1^st^ to 9^th^ grades from 20 schools (*n* = 7 condemning approach, *n* = 7 empathy-raising approach, and *n* = 6 combined approach), school personnel had reported on (at least some steps) of the implemented targeted intervention. In Supplemental Material 1, it is more precisely described how this final sample of 274 victims was obtained. For instance, if victims were repeatedly included in a targeted intervention (e.g., if their cases were handled in an intervention in both school years) only the information of the *first targeted intervention* was retained - to prevent interdependence of observations. Remarkably, in schools implementing the condemning approach, victims’ cases required *repeated interventions* in about 10% of the cases, whereas in combined schools this was never required. The average number of victims whose bullies received a targeted intervention per school across the two academic years was 13.70 (*SD* = 11.97), and ranged from 4–47. A total of 113 victims had bullies who received a condemning approach, 117 victims had bullies who received an empathy-raising approach, and 44 victims had bullies who received a combined approach. Out of the 274 victims, 40.5% identified as boy and 56.6% identified as girl. The victims’ mean age at the year of the intervention was *M* = 11.95 years, *SD* = 1.89, with an age range of 7.95–15.59 years.

#### Survey Data Procedure and Participants

Survey data was collected as part of the Challenge project three times in each academic school year (T1 = October, T2 = January, T3 = April). Students were followed for two consecutive school years. Only students who received parental consent and gave their assent participated in these surveys. Across waves, online questionnaires were administered under the supervision of classroom teachers, who had been carefully instructed about the data-collection. Confidentiality of students’ answers was guaranteed and they were told that they could opt out at any time. Topics of the questionnaire included frequency of bullying victimization, psychological adjustment, and cognitions about bullying (e.g., self-blame).

The survey data enabled examining the longer-term development of victimization. Specifically, it enabled testing whether victims whose cases were handled in a targeted intervention would be more likely to decrease in victimization from the start of (or halfway through) the school year until the end of the school year, as compared to victims whose cases were not handled in an intervention. Therefore, for each school year, students were only selected if they indicated in the survey’s general or specific victimization items at T1 *or* T2 that they had been victimized (for more information on the items, see Measures section below). Out of the *n* = 6265 students in year 2020–2021 (year 1), *n* = 3126 students indicated at T1 or T2 on at least one of the victimization items that they had been victimized at least once or twice the past few months. Out of the *n* = 5873 students in year 2021–2022 (year 2), *n* = 2722 students indicated at T1 or T2 on at least one of the victimization items that they had been victimized at least once or twice the past few months. A total of *n* = 1537 students reported being victimized in both years.

### Measures

#### Short-Term Changes in Victimization

During the fifth and sixth meeting (see Fig. 1), victims were asked by their teacher whether their victimization had (1) stopped, (2) decreased, (3) remained the same, or (4) increased. Some victims were present during both meetings. It was decided to use the information of the fifth meeting, because it was expected victims would be most honest about the situation in the absence of their bullies; and victims were not always present in the final follow-up meeting with the bullies. Based on previous research (Garandeau et al., 2014) it was anticipated that the number of victims indicating that the victimization had remained the same or increased would be very small. Therefore, the variable was categorized as follows: 1 = victimization stopped, 0 = victimization diminished, remained the same or increased.

#### Self-Reported Victimization

Survey data was used to examine longer-term changes in victimization. At the beginning of the survey, students were presented with a definition of bullying, emphasizing aspects of intentionality, repetition, and power imbalance. Next, they reported the frequency with which they had been bullied in general (this item was only used to determine who was victimized at T1 and T2) and next, how they had been bullied in five specific ways during the past couple of months, including: “I was left with no attention or outside all things or all company by my classmates”, “I was hit, kicked or pushed”, “Other pupils tried to get others hating me by gossiping and telling lies about me”, “I was called nasty names or laughed in my face or hurt by insults” and “I was bullied online” (Solberg & Olweus, 2003). The answers were given on a 5-point Likert scale, with 1 = not at all, 2 = once or twice, 3 = 2–3 times a month, 4 = once a week, and 5 = several times a week. The reliability of this scale varied from ω = 0.73–0.80 across waves across year 1 and year 2. The scale has been shown to be valid in previous work using the same dataset (Laninga-Wijnen et al., 2024b). If students had missing data or did not indicate that they were victimized at T1, *and* if the targeted intervention had taken place *after* T2, their scores at T2 were used as baseline.

#### Targeted Intervention Type

Two dummy variables were created to assess the targeted intervention approach: (1) a dummy for the *empathy-raising approach* versus other approaches, (2) a dummy for the *combined approach* versus the other approaches. Thus, the condemning approach was the reference category. This reference category was chosen because this traditional approach has been investigated most consistently in previous work and because this method has been the most often used and advised approach across anti-bullying interventions (Olweus, 1996).

#### Control

A dummy was created for no intervention (1) versus intervention (0). Thus, students whose cases were handled in a targeted intervention were the reference category.

#### Biological sex

We synchronized information from the survey data and targeted intervention data to obtain students’ biological sex. It was coded as 1 = boy, 0 = girl.

#### School level

Level of schooling was coded with 1 = secondary school and 0 = primary school. It was assessed on the individual level because within combined schools the level may vary (either 1 or 0) per student. Moreover, given small sample size of victims per school, it would be too demanding to run multi-level analyses. Additionally, the intraclass correlation of victimization was very low (ICC = 0.017), showing that there was not much variance to be explained at the school-level.

#### Duration of victimization

In the first targeted intervention meeting, victims were asked how long the bullying had been going on. The proportions of durations mentioned were: 0 = a week or two (16.5%), 1 = one to two months (19.6%), 2 = two to six months (28.2%), 3 = six to 12 months (12.4%), 4 = more than a year (22.3%).

### Analytic Strategy

The current study has been pre-registered, and all deviations of the pre-registration have been reported in Supplemental Material 5. Most deviations pertain to the steps taken to retrieve the final dataset that was used for analyses. To examine the short- *and* long-term effectiveness of targeted interventions, data from the targeted intervention subproject *and* the survey subproject were combined in various steps. These steps will now be shortly explained, after which the analytic approaches are introduced and justified.

#### Steps taken to create dataset

For each school year separately (i.e., two datasets), several steps were taken to create the final dataset for analyses. Step 1 was to combine targeted intervention data with survey data; Step 2 was to impute data using Multiple Imputation; Step 3 - for longer-term analyses- was to identify a control group using propensity score matching, and Step 4 was to merge files of the two academic school years (for each imputed dataset).

##### Step 1. Combining targeted intervention data and survey data

First, one dataset was created for each academic year, including all students who (1) indicated to be victimized on the survey, or (2) were enrolled in a targeted intervention, or (3) both. For this latter category, targeted intervention data was combined to survey data based on an ID-number that was provided by teachers (for more thorough information, see Supplemental Material 2). It was only possible to combine targeted intervention data with survey data for students (A) who had consent to participate in the survey data-collection (in the full sample, 70.8% of students had parental consent), and (B) whose teachers reported a valid ID-number that enabled combining datasets.

For *n* = 127 targeted intervention victims these criteria were not fulfilled and therefore it was not possible to combine their targeted intervention data with survey data. They were however kept in the dataset, because there was information on the short-term effectiveness of targeted interventions for these students. For the other 147 victims included in a targeted intervention, targeted intervention data could be combined with survey data. From these 147 victims, *n* = 20 victims had been absent at both the T1 and T2 survey administration and hence had missing values on survey-reported victimization. Therefore, these victims were only included in evaluating the longer-term effectiveness if imputed survey values suggested that they had been victimized.

##### Step 2. Missing data imputation

Both survey data and targeted intervention data had a relatively large proportion of missing data, warranting imputation (van Ginkel et al., 2020). Missing data in the current study stemmed from various sources. For instance, for *n* = 76 students, teachers did not document the outcome of the targeted intervention on the follow up meeting. Moreover, around 50.0% of students did not participate during one or more waves of the survey data-collection (for more information, see Supplemental Material 3). An imputation technique was applied that is the best response to missing data stemming from various sources in both dependent and independent variables, and that can partly remove bias that may occur due to MAR or MNAR: Multiple Imputation (Van Ginkel et al., 2020) in the MICE package (Austin & van Buuren, 2023), implemented in R Version 4.2.2. Depending on the nature of each variable, Predictive Mean Matching (continuous or categorical with ordered categories), Polynomial Logistic Regression (categorical with nominal categories) or Binary Logistic Regression (binary), were used to generate 50 imputed datasets, and this was done for each school year separately. Thus, for each school year, 50 imputed datasets were generated. A matrix of predictor, outcome, and auxiliary variables is available upon request. Supplemental Material 3 also explains how auxiliary variables have been assessed.

##### Step 3. Selection of control group for longer-term analyses: propensity score matching

To compare the longer-term development of victimization among students enrolled in an intervention vs. those who were not, survey data were evaluated. A great number of students reported that they had been victimized at least once or twice on at least one specific victimization item at T1 and/or T2 on the survey (*n* = 4311 unique students in total across the two years). Yet, only a very small part of them had actually been enrolled in a targeted intervention (*n* = 274; 6.4%). Moreover, from this targeted intervention group, it was only possible to examine longer-term change in victimization for those who did have consent on to participate in the survey, which was *n* = 147 at most, but the exact number of students varied across imputations because it depended on how many out of 20 students with missing data on survey-reported victimization were imputed in such a way that they were considered victim. This implies that when comparing intervention victims to control victims, the group of control victims would be overly large, yielding issues with increased Type I error rates because the larger group may have a disproportionate influence on the results. Therefore, after combining and imputing targeted intervention data and survey data, propensity score matching was applied to retrieve an equally populated intervention and control group. Specifically, within each school year, on all 50 imputed datasets, propensity score matching enabled to select a group of students who had not received an intervention (control group) but who were comparable to the intervention group in terms of biological sex, age, school level (primary/secondary), anxiety, peer acceptance, and self- and peer-reported victimization at the start of the school year. These variables were selected because previous work found them to relate to students’ disclosing of the victimization, or to peers’ efforts to end victimization (e.g., by telling the teacher about the situation; Rambaran et al., 2021; Shaw et al., 2019). Thus, these items may predict whether students’ cases will be handled in a targeted intervention or not. Details regarding matching as well as balance tables (pooled over various datasets) for year 1 and year 2 respectively are reported in Supplemental Material 4 (Table S1). Adjusted standardized mean differences were all well below 0.10, indicating that the control group and intervention group were highly comparable in characteristics used for matching. A matching control was found for all intervention students, thus no intervention student had to be excluded.

##### Step 4. Merging data of academic years

After selecting - for each imputed datafile - the control group, the files of the two academic years were merged, resulting in 50 imputed datasets involving an intervention group of *n* = 274 students and a control group varying from 129–144 victim cases (because for every imputed dataset, the number of victims could differ depending on the survey values imputed for victimization). Note that the size of the control group matched the size of the intervention group for the *longer-term* analyses, which only included students who had consent for survey and who indicated (or were imputed) to be victimized at T1 or T2. A total of 32.9% of the cases were handled with the condemning approach, while 41.6 and 25.5% were handled with the empathy-raising approach and combined approach respectively. Furthermore, 54.4% of the students in these imputed files identified as girl, and 35.7% were from secondary schools.

All analyses described below were conducted on the 50 imputed datasets separately, after which the parameter estimates, fit measures, and inference tests were pooled to obtain a final result.

#### Analyses for Short-Term Effectiveness

To examine the *short-term effectiveness* of targeted interventions in diminishing victimization, several steps were taken. First, the percentages of cases was evaluated in which the victimization stopped, decreased, remained the same, or increased - as assessed in the follow up meeting of targeted interventions. Next, a series of logistic regression models were run in R Version 4.2.2. The first model tested the effects of main predictors (approach taken in targeted intervention) on the odds that the victimization had stopped or not at follow up. In the second model, main effects were estimated in conjunction with the covariates (biological sex, school level, and duration of the victimization). In these analyses, the condemning approach, primary schools, and girls were the reference groups (coded as 0). Model 2 explored the relative short-term effectiveness of various approaches. In the next models, interaction effects were evaluated between various intervention approaches and (1) the duration of victimization, and (2) school level. Given that duration of victimization was a continuous variable, it was grand-mean centered before calculating interaction terms.

#### Analyses for Longer-Term Effectiveness

Next, the *longer-term effectiveness* of targeted interventions was examined on a sub-dataset of survey-consenting students enrolled in an intervention and an equally populated and matched control group. Specifically, it was examined whether students whose cases were handled in a targeted intervention reported a stronger within-person decline in survey-reported victimization than students whose cases were not handled in a targeted intervention. To this end, a series of Latent Change Score Models (LCSM) was estimated. LCSM combine strengths of path modeling and latent variable modeling (Kievit et al., 2018). Latent variables (multiple indicator models) were used to account for measurement errors in the observed items and to increase power and validity (Kievit et al., 2018). Change over two time points (y3 – y1) was modeled with a latent change score (Δy). The LCS models were estimated using the lavaan package (Rosseel, 2012), using the Maximum Likelihood estimator. Before testing the hypotheses, it was tested for longitudinal measurement invariance in the sample of survey-consenting intervention and control victims – pooled scalar fit was tenable (CFI = 1.00, TLI = 1.025, RMSEA < 0.001, SRMR = 0.014). Then, the pooled average degree of within-person change in victimization from the start till the end of the school year was estimated, referred to as Δy.

As a next step, several LCSM were run to test the hypotheses. In Model 1, it was tested whether students whose cases were handled in a targeted interventions experienced a stronger within-person decline in victimization as compared to their matched control students. To this end, the model included the dummy for the control group (1) versus the intervention group (0) as predictor of latent change (from T1–T3) in victimization. In Model 2, to explore the relative effectiveness of different targeted intervention approaches, an LCSM was estimated, which included dummies for being enrolled in the Combined approach (1) versus the rest (0) and being enrolled in the Empathy-raising approach (1) versus the rest as predictors of change in victimization. Only if the model increased in fit, effects of these dummies were interpreted. In Model 3, it was examined whether intervention effects would hold after including covariates (biological sex and school level).

## Results

### Short-Term Effectiveness of Targeted Interventions

Before examining short-term effectiveness of targeted interventions, some descriptive findings were evaluated. During the follow-up meeting with the teacher, the majority of victims indicated that the bullying had improved, i.e., that it had been stopped (61.6%) or reduced (26.6%). For 11.1% of the victims, the bullying had remained the same, whereas for 0.7% of the victims, the bullying had increased. In Table 1, victims’ evaluations on whether the bullying had stopped, decreased, remained the same/increased, have been split out for each method (condemning, empathy-raising, and combined). The percentage of cases in which the situation of the victim improved (bullying stopped *or* decreased) was highest for the Combined approach (91.6%), followed by the Empathy-raising approach (88.3%) and it was lowest for the Condemning approach (86.7%).

**Table 1:** Cross-tabulations comparing approaches on short-term changes in victimization (*N* = 274)

	Condemning (*n* = 113)	Empathy-raising(*n* = 117)	Combined (*n* = 44)
Stopped	64.8%	55.3%	70.0%
Decreased	21.9%	33.0%	21.6%
Remained the same/increased	13.3%	11.7%	8.4%

Logistic regression models were run to examine the relative effectiveness of various approaches in stopping victimization (Table 2). The empathy-raising and combined approach did not significantly differ from the condemning approach in predicting the likelihood that victimization ceased (Model 1). The duration of the bullying was unrelated to victimization cessation at follow up, nor did this duration moderate the extent to which the combined and empathy-raising (vs. condemning) approaches stopped victimization (Model 3, Table 2). Targeted interventions were 1.9 times more likely to stop victimization for boys than for girls, and 2.3 times more likely to stop victimization for secondary school students than for primary school students. School level (primary vs. secondary school) did not significantly moderate the extent to which combined and empathy-raising (vs. condemning) approaches related to ceased victimization (Model 4, Table 2).

**Table 2:** Logistic regression models predicting the likelihood that the victimization had stopped (1) vs not (0) after the targeted intervention (*n* = 274)

	Model 1: Main effects			Model 2: Main & control effects			Model 3: Moderation bullying duration			Model 4: Moderation school level		
	*Est(SE)*	*p*	*OR*	*Est(SE)*	*p*	*OR*	*Est(SE)*	*p*	*OR*	*B* (SE)	*p*	*OR*
Empathy-raising	−0.399 (0.301)	0.093	0.671	−0.050 (0.362)	0.401	0.952	−0.042 (0.362)	0.454	0.959	−0.438 (0.450)	0.416	0.645
Combined	0.241 (0.435)	0.291	1.272	0.437 (0.491)	0.188	1.548	0.424 (0.495)	0.197	1.528	0.230 (0.573)	0.344	1.258
Duration of victimization				−0.106 (0.134)	0.216	0.899	−0.100 (0.284)	0.368	0.901	−0.100 (0.135)	0.230	0.904
School level (0 = primary)				**0.825** (**0.374)**	**0.014**	2.281	**0.823** (**0.377)**	**0.016**	**2.277**	0.474 (0.462)	0.153	1.607
Boy				**0.631** (**0.356)**	**0.040**	1.879	**0.623** (**0.360)**	**0.044**	**1.865**	0.595 (0.362)	0.052	1.813
Interaction with empathy-raising							−0.042 (0.205)	0.419	0.960	0.872 (0.703)	0.110	2.39
Interaction with combined							−0.220 (0.468)	0.319	0.802	0.255 (1.076)	0.406	1.290
Intercept	0.613 (0.226)	0.004	1.847	−0.149 (0.367)	0.343	0.861	−0.153 (0.368)	0.339	0.858	0.089 (0.421)	0.421	1.093

In Model 3, interactions were tested between victimization duration and approaches (empathy-raising and combined), and in Model 4, interactions were tested between school level and approaches

Bolded estimates are significant with *p* < *0*.05

### Longer-Term Development of Victimization Following Targeted Interventions

Before examining the effect of targeted interventions on longer-term within-person changes in victimization, a *univariate* multiple indicator LCSM explored the *average* changes in victimization from the start till the end of the school year within the group of survey-consenting intervention and control victims (Fig. 2). The latent constructs for victimization (VIC) included a baseline measure (mostly assessed in October; referred to as VIC T1) and an end-of-the-year assessment (VIC T3), each measured using five indicators (V1T1, etc.). The change in victimization over the two time points was modeled as a latent change score (ΔVIC). Means were included in the model by adding a constant term (value 1.00) as symbolized by the triangle in the path model. The mean changes were estimated by tracing all paths from the triangle to the latent change score (Δx). To calculate average change in victimization (*m* Δ VIC), the path from the triangle to ΔVIC was summed, as well as the path from the triangle to ΔVIC via VIC T1, as indicated by the dashed paths in Fig. 2. The model fit of this univariate LCS was adequate, with (CFI = 1.000, TLI = 1.061, RMSEA < 0.001, SRMR = 0.051). The estimated means indicated that students - on average - were stable in victimization across the school year, with *M* = 2.284 and *M* = 2.177 on T1 and T3 respectively (*m Δ* VIC = −0.107, *SE* = 0.076, *p* = 0.164). Higher victimization levels at the start of the school year related to a smaller increase in victimization throughout the academic year (*Est* Vict 1 → Δ Vict = −0.180, *SE* = 0.082, *p* = 0.031).

Then, in three LCSM, the role of targeted interventions in the longer-term development of victimization was examined. Model 1 included the dummy for the intervention (Control = 0) versus control (Control = 1) group. The model fitted the data well (pooled fit statistics of RMSEA = 0.000, CFI = 1.000, TLI = 1.098, SRMR = 0.048). In contrast to the hypothesis, victims whose cases were handled in a targeted intervention did not decline more strongly in victimization as compared to victims whose cases were *not* handled in a targeted intervention (pooled *Est* = −0.109, *SE* = 0.143, *p* = 0.447; Table 3). The Relative Increase in Variance (RIV) of this pooled estimate was 0.992, indicating that between-imputation variance did not exceed within-imputation variance, suggesting that the estimates from the different imputations are consistent.

**Table 3:** Results of three LCSM on longer-term changes in self-reported victimization (*n* varies from 258–286 per sub-dataset)

	Model 1		Model 2		Model 3	
	Est. (SE)	*p*	Est. (SE)	*p*	Est. (SE)	*p*
*m* Δ Vict	−0.107 (0.074)	0.149	−0.107 (0.070)	0.127	−0.107 (0.068)	0.116
I Vict 1	**2.285** (**0.109)**	**<0.001**	**2.285** (**0.104)**	**<0.001**	**2.285** (**0.100)**	**<0.001**
I Δ Vict	0.359 (0.208)	0.086	**0.476** (**0.217)**	**0.029**	**0.493** (**0.248)**	**0.048**
Vict 1 → Δ Vict	−**0.179** (**0.079)**	**0.025**	−**0.181** (**0.075)**	**0.017**	−**0.184** (**0.073)**	**0.013**
Control → Δ Vict	−0.109 (0.143)	0.447	−0.119 (0.136)	0.383	−0.120 (0.132)	0.365
Empathy-raising → Δ Vict			−0.202 (0.158)	0.203	−0.233 (0.166)	0.160
Combined → Δ Vict			−0.138 (0.173)	0.425	−0.166 (0.177)	0.349
Boy → Δ Vict					0.001 (0.132)	0.992
School level → Δ Vict					−0.072 (0.149)	0.631
R^2^	0.067		0.082		0.084	

I Vict 1 is the intercept of the latent factor of self-reported victimization at the start of the school year. I Δ Vict is the conditional intercept for the latent change score factor of victimization. In Model 1, all targeted interventions are the reference group. In Model 2, the Condemning approach is the reference group. The average duration between the intervention starting point and the end-of-year assessment of victimization was 131 days

Bolded estimates are significant with *p* < 0.05

In Model 2, two dummies were added: for 1) the combined approach versus the rest and 2) the empathy-raising approach versus the rest. Therefore, the condemning approach became the reference group in this model. The model fit was slightly better as compared to the model only including control vs. intervention group (ΔRMSEA = −0.000, ΔTLI = 0.004, ΔSRMR = −0.005). The combined approach and empathy-raising approach did not relate to a significantly stronger decline in victimization as compared to the condemning approach (pooled *Est* = −0.138, *SE* = 0.173, *p* = 0.425 and pooled *Est* = −0.202, *SE* = 0.158, *p* = 0.203, respectively), and the victims whose cases were handled with a condemning targeted intervention did not experience a stronger decline in victimization than control students (pooled *Est* = 0.119, *SE* = 0.136, *p* = 0.383). The negative estimates suggest that victims whose cases were handled with a condemning approach experienced the smallest decrease in victimization across the academic year, even though none of these differences reached statistical significance. The RIVs of pooled estimates varied from 0.737–0.962, thus between-imputation variance did not exceed within-imputation variance. The model explained 8.2% of the variation in latent change in victimization. In Model 3, the inclusion of biological sex and school level slightly improved model fit in terms of TLI and SRMR (ΔTLI = 0.066, ΔSRMR = −0.003), even though these covariates were unrelated to changes in victimization (pooled *Est* = 0.001, *SE* = 0.132, *p* = 0.992 and pooled *Est* = −0.072, *SE* = 0.149, *p* = 0.631 respectively, with RIV = 0.789 and RIV = 0.767).

### Sensitivity Analyses

All decisions regarding sample size, data exclusions, and measures have been reported. Three sensitivity analyses have been conducted, and findings available upon request. First, models testing the short-term effectiveness were analyzed again while applying full information maximum likelihood rather than multiple imputation, and results remained highly similar. Second, a simpler matching procedure was used, with four variables (school-level, gender, self- and peer-reported victimization) and another set of LCSM was run on the control sample retrieved using that simplified matching procedure. Again, findings were almost identical. Lastly, LCSM were run while including the duration in days between the starting date of the targeted intervention and the end-of-the-year assessment of survey-reported victimization, to account for potential maturity threats (e.g., that students whose cases were handled later in the year may be subjected to more persistent bullies). No effect emerged of the duration in days, and the other effects in the LCSM remained the same.

## Discussion

Teachers often wonder what they can do if - despite prevention efforts - cases of bullying victimization come to their attention (Salmivalli et al., 2025). Many anti-bullying programs include not only preventive elements, but also guidelines for *targeted interventions* (e.g., series of discussions with victims and bullying perpetrators). Yet, previous evaluation trials tested the effectiveness anti-bullying programs as a whole, rather than separating prevention and targeted intervention effects (Hensums et al., 2023), with the exception of a few targeted intervention studies (e.g., Garandeau et al., 2014). The present targeted intervention study tested the relative effectiveness of three approaches that teachers can take when intervening in systematic cases of bullying victimization: 1) enhancing empathy for victims in bullies, 2) condemning bullying behaviors, and 3) combining these approaches. It builds on prior targeted intervention research by incorporating ecological momentary assessments, evaluating both short- and long-term outcomes, and including a control group of victims whose cases were not handled in a targeted intervention. On the short-term, 88.2% of the victims indicated that their situation had been improved, meaning that the victimization had *decreased or ceased* as a result of the intervention. The combined approach seemed to be the “best bet”, because the percentage of *fully ceased* victimization was highest (70.0%), even though differences between approaches did not reach statistical significance. In the longer-term, victims whose cases were handled in a targeted intervention were equally stable in victimization as compared to the control group - irrespective of the approach taken (condemning, empathy-raising, combined). Thus, despite initial improvements, on the longer term, systematically victimized youth do not seem to escape their plight.

### The Short-Term and Longer-Term Effectiveness of Targeted Interventions: Good and Bad News

The findings on the short-term effectiveness of targeted interventions present encouraging results. Consistent with earlier targeted intervention studies (e.g., Garandeau et al., 2014), the use of ecological momentary assessments in this study revealed high success rates in reducing or ceasing victimization at follow up, ranging from 86.7% for the condemning approach to 91.6% for the combined approach. Although no significant differences emerged between the condemning, empathy-raising, and combined approach in achieving full cessation of victimization, the results seem to support prior findings (e.g., Johander et al., 2022) suggesting the combined approach may be especially effective: 70.0% of victims in the combined approach reported cessation, compared to 55.3% in the empathy-raising approach, and 64.8% in the condemning approach. Furthermore, victims whose cases were handled using the combined approach were not victimized again in a way that called for a targeted intervention - in other words, they did not show up in new cases later in the school year, unlike those whose bullies received the condemning or empathy-raising intervention. This observation aligns with prior research indicating that disciplinary methods (e.g., condemning) are most effective when applied within a supportive context (Burger et al., 2022).

However, the promising short-term outcomes are tempered by less optimistic findings regarding longer-term change in victimization levels. Self-reported victimization remained relatively stable across the school year, with no stronger reduction in victimization among cases addressed by targeted interventions compared to cases not addressed, regardless of intervention type. Notably, this does not necessarily suggest that the interventions failed to alter the behavior of the perpetrators. Rather, the findings indicate that students who experienced high levels of victimization at the beginning of the school year continued to report similar levels at the years’ end regardless of whether their situation had been addressed in an intervention at some point that year.

The persistence in victimization on the longer term may have multiple underlying causes. First, the bullies may have resumed the bullying after the follow up meeting, or the victims may have become targets of new bullies. Consistent with prior research, specific victim characteristics or peer rejection may render certain students vulnerable to ongoing victimization, creating a self-perpetuating cycle that is challenging for educators to disrupt (Farrington & Baldry, 2010; Kaufman et al., 2020). Additionally, the sample contained quite severely victimized students, with average victimization scores of 2.284 at T1 (or T2) and 2.177 at T3 on a 1–5 scale. This concentration of high-severity cases may indicate that, for frequently victimized students, breaking the cycle of bullying is particularly difficult—a finding that does not preclude targeted interventions from being effective in less severe cases. Previous research supports the notion that interventions are less effective for highly victimized students (Garandeau et al., 2014; Johander et al., 2024), possibly due to the escalation of the situation and the development of fixed interaction patterns between perpetrators and their victims (Gardner et al., 2019), which underlines the importance of early intervention. Severely victimized students may also face multiple perpetrators, making it harder for teachers to stop all of them (Rigby & Barnes, 2002). Second, is also plausible that this specific group of victims exhibited a higher tendency to perceive negative social interactions as bullying, potentially reflecting a hostile attribution bias where ambiguous social situations are interpreted as bullying (Kellij et al., 2022). This may explain why they continue perceiving themselves as victim of bullying, even if bullying did actually cease. A final reason why no significant effect was detected could be a statistical one: the sample in which the longer-term development of victimization was evaluated was rather small, in particular for students in the combined approach (*n* = 44 for the short-term analyses, and *n* = 34 on average for the longer-term analyses). Despite all efforts of using state-of-the-art imputation techniques with numerous auxiliary variables and thorough matching procedures, this statistical reason should not be overseen.

The current study findings should not be interpreted as evidence that targeted interventions are ineffective or that implementing such interventions is equivalent to taking no action. Instead, the results highlight the need for further research in this field. However, as evidenced in this study, achieving adequate sample sizes remains challenging. Future studies should prioritize examining the impact of targeted interventions on bullying perpetrators, comparing their efficacy in reducing or stopping bullying behaviors in both the short-term and longer-term. Additionally, research should investigate which characteristics of bully perpetrators may influence the effectiveness of various intervention strategies (Farrington & Baldry, 2010). Another essential area for future investigation involves determining whether persistent victimization, despite intervention, is due to continued harassment by the same individuals or the emergence of new perpetrators. Although this was an initial goal of this study, extensive missing data—exceeding 80% in some classroom peer networks—precluded reliable analyses (Zandberg & Huisman, 2019).

Future research should aim to refine teachers’ strategies in targeted interventions, specifically in conversations with victims (step 1, Fig. 1) and potential supporters (step 4, Fig. 1). Current guidelines generally advise teachers to listen impartially to victims’ accounts, yet there may be additional effective approaches. For instance, teachers might explore victims’ causal attributions, and emphasize to victims that they should not blame themselves for their situation, as self-blame has been found to relate to continued victimization (Schacter & Juvonen, 2017). Further investigation is also needed into interventions involving peer supporters. While past research indicates that defender behaviors, such as those fostered in support groups, can have positive short-term effects on daily mood (Laninga‐Wijnen et al., 2024a), they have not been shown to reduce victimization (Laninga-Wijnen et al., 2023). It is essential to examine which specific types of defending and support strategies might lead to the positive, sustained outcomes researchers and school personnel aim to achieve.

### Covariates: School Level, Biological Sex, and Duration of Victimization

Analysis of covariates revealed that victimization was more likely to decrease in the short term among secondary school students than among primary school students. This finding is somewhat surprising, considering the developmental period of adolescence, when commands and requests from adult authorities are not always well-taken (Veenstra & Laninga-Wijnen, 2022). The finding also diverges from prior studies, which reported that targeted interventions were less effective in secondary schools than in primary schools (Johander et al., 2021, 2022). Additionally, meta-analyses suggest that whole-school anti-bullying programs are more effective among younger students than older ones (Hensums et al., 2023). A possible explanation for the stronger short-term reduction in victimization among secondary school students may be that older students are more responsive to targeted interventions due to increased cognitive maturity and social awareness, which may help them better recognize and respond to intervention strategies (Laninga-Wijnen and Veenstra, 2022).

Furthermore, findings indicate that victimized boys were more likely to report that their victimization had stopped on the short-term. Previous work has shown that boys are more likely to decrease in victimization over time even in the absence of interventions (Laninga-Wijnen et al., in press) - it could be that bullying situations with boys resolve more naturally than those with girls. However, in the longer-term, this study found no significant effects of biological sex or school level on victimization, indicating that any initial gains among secondary school students and boys may not be sustained over time. This finding does align with previous work on the longer-term effectiveness of targeted interventions (Johander et al., 2021). Duration of victimization was unrelated to short-term cessation, which does not align with a previous study (Garandeau et al., 2014). It is possible that the mobile phone application helped teachers to better adhere to guidelines for the targeted interventions, ensuring greater effectiveness irrespective of bullying persistence.

### Strengths and Limitations

The current study has several strengths, including the use of ecological momentary assessments, which prevents memory bias, ensures that teachers are aware of guidelines for the approach they were assigned to, and safeguards ecological validity. Moreover, this study was the first to include the combined approach as condition that could be compared to condemning and empathy-raising approaches - previous work examined the extent to which teachers reported to use either condemning or empathy-raising strategies, without explicitly instructing and training teachers in how these approaches could be optimally combined. Furthermore, this study did not only examine the short-term but also the longer-term development of victimization in students whose cases were handled in targeted interventions. Including a control group whose cases were not addressed in an intervention helped determine whether the observed changes in the intervention group were due to the intervention itself or simply natural changes that would have happened anyway.

Despite these strengths, limitations should be acknowledged. The first limitation is the high amount of missingness in the data, which was addressed in the best possible way by implementing cutting-edge multiple imputation techniques that are robust against normality violations and can even handle missingness not completely at random (Van Ginkel et al., 2020). Although from a conceptual perspective it may seem inappropriate to impute information that decides whether students are seen as “victim” or for instance, students’ biological sex, from a statistical perspective, the model used for multiple imputation does not have to be a conceptually meaningful model. Multiple imputation is only used to preserve the relationships and structures within the data while imputing missing values with similar properties. Any variable that correlates with another variable containing missing data can serve as a predictor in the imputation model, regardless of whether the relationship is conceptually meaningful (van Ginkel et al., 2020). Thus, missing data was handled in advanced ways.

A second limitation is the relatively small sample size, although this aligns with instructions for school personnel to report their interventions in *systematic cases of bullying*. Moreover, the low prevalence of targeted interventions is an interesting finding in and of itself. In the sample of 6265 students, the proportion of students reporting that they were repeatedly (2–3 times a month or more often) bullied varied from 11.6–14.7% across measurement waves, which aligns with previous work (Biswas et al., 2022; Bjereld et al., 2020). However, as also shown by previous research (e.g., Garandeau et al., 2014; Haataja et al., 2016) it is a minority of bullying cases that end up in targeted interventions. Such a low prevalence of interventions targeting systematic bullying can probably be explained by students not disclosing their victimization (Van der Ploeg et al., 2022), teachers doing something else, such as intervening in victimization on the spot, rather than resorting to a series of discussions that require more time and effort (Salmivalli et al., 2025), or teachers not intervening at all, because they feel incompetent or do not perceive the situation as severe (van Aalst et al., 2024). The prevalence of intervention cases in the current study aligns with (and is even slightly higher than) other targeted intervention studies that used similar instructions (Garandeau et al., 2014; Haataja et al., 2016). Thus, the low sample size suggests that the proportion of cases intervened in an evidence-based way is disappointingly low, even when teachers are provided with detailed guidelines to do that. A third limitation is that the focus on victimization precludes insights in what happens to those who are actually targeted in the interventions: the perpetrators. Further studies using the same data will examine the effectiveness of targeted interventions from the bullies’ perspective - an investigation that poses additional challenges that could not be addressed in the current study (e.g., the need to convert to peer reports because of social desirability in self-reported bullying, bullies being nested in multiple incidents; Košir et al., 2020). Yet, the ultimate aim of targeted interventions is to help victims escape their plight, and therefore examining the role of targeted interventions in changes in victimization is a worthwhile first step.

A fourth limitation regarding the *short-term* effectiveness, is that school personnel implementing the targeted intervention were the ones asking at follow up about its effectiveness, which may have caused social desirability issues: students may be aware of the efforts put in the situation, and they may feel reluctant to disclose that these efforts actually had been ineffective. This may have been an underlying reason for the discrepancy between the short- and longer-term effects, too.

Fifth, the current study did not consider to which extent victimized students received help from their classmates in other forms, such as via peer defending (which typically is encouraged in step 4 of targeted interventions). Data on step 4 (e.g., whether it was implemented, and if so, how) was very scarce, and based on previous research, it was not anticipated that peer defending would decrease peer victimization over time (Laninga-Wijnen et al., 2023; Van der Ploeg et al., 2016), which was why this was not part of the pre-registered plan of the study. Future work is encouraged however to examine the potential effectiveness of peer intervention in systematic cases of bullying. Moreover, teacher characteristics (e.g., conscientiousness, competence in handling victimization) may have mattered for the reporting of the steps, or for intervention effectiveness. In the current study, an intervention team consisted of at least three teachers (or other school personnel), so that the reporting and intervention implementation was not fully dependent on (characteristics of) one person. Yet, there are large variations in the extent to which all steps were reported. Future work is encouraged to examine the role of teacher characteristics in the effectiveness of targeted interventions, and to evaluate to which extent fidelity to all steps of targeted intervention may affect effectiveness.

## Conclusion

School bullying is a significant societal concern with detrimental effects on all students. It therefore is of utmost importance that teachers know how to address cases of victimization that come to their attention- in other words, how to implement a *targeted intervention*. The current study compared three approaches teachers can take when talking to bullying perpetrators: condemning the bullying, raising empathy for victims, or both. The first important finding of this study is that targeted interventions are applied to only a small percentage of systematically targeted students, even when teachers are provided with detailed guidelines (i.e., in a mobile phone application) on how to do that. This is unfortunate, as the current study showed that targeted interventions are effective at reducing or ceasing victimization at the short-term. Therefore, teachers should encourage students’ openness about victimization and, if cases of bullying come to their attention, *systematically intervene* in it (rather than “on-the-spot”) by organizing a series of discussions with students involved. This study suggests that during these meetings, providing a combined message (i.e., condemning the bullying behavior *and* attempting to raise empathy for victims) to bullying perpetrators may be most beneficial for reducing victimization on the short-term. It is promising that these interventions work (even especially) in the development period of adolescence, as previous work on anti-bullying prevention in adolescence revealed no or iatrogenic effects (Yeager et al., 2018). Importantly, in the longer-term, victimized students whose cases were handled in targeted interventions did not seem to escape their plight and seemed to remain targets of victimization - either by new or the same bullies. This illustrates the need for teachers to carefully monitor victimization in the cases in which they intervened in throughout the school year, for instance by not only planning an evaluation after two weeks, but also after a few months - so that new actions can be taken if required. It is important that the field of bullying research shifts its focus from intervention success to the examination of intervention failure - both on the short *and* longer-term. More research should be conducted on the role of parents and peers, who can also intervene in bullying, and future studies are encouraged to identify factors associated with intervention failure and success. Only this way, key obstacles towards targeted intervention success will be identified and addressed, which will significantly improve the school lives of victimized students.

## Supplementary information

Supplementary Material

## Data Availability

The datasets generated and/or analyzed during the current study are not publicly available but are available from the last author on reasonable request, with necessary safeguard to guarantee anonymity. The analytic code for reproducing results is available on https://osf.io/94tvg/files/osfstorage.
