# Supplementary material for: Do Targeted Interventions Diminish Victimization? Testing the Short- and Longer-term Effectiveness of Condemning, Empathy-Raising, and Combined Approaches
Source: J Youth Adolesc. 2025 Apr 15;54(7):1659–76. doi: 10.1007/s10964-025-02173-0 (PMC12246010; doi:10.1007/s10964-025-02173-0)
Supplement: Supplementary file 1 — Supplementary Material [file 10964_2025_2173_MOESM1_ESM.docx]

**Supplementary Material 1 - Determination of number of victims for which targeted intervention information had been reported by teachers**

Teachers were asked to report each incident for which they implemented a targeted intervention. Incidents could involve multiple bullies and multiple victims. In total, teachers reported on the steps they took for 288 incidents. However, when carefully reviewing the description of incidents provided by school personnel, eight of them were discussions concerning mutual fight(s) rather than bullying. Furthermore, based on this same description it was noticed that four cases were ‘try out’ cases, which teachers had inserted to try out the app. Moreover, six cases appeared to be duplicates, i.e., the same incident was entered in the system twice. Therefore, these were erased from the data. The remaining 270 incidents involved 314 victimized students, because in *n* = 20 incidents, multiple victims were reported (on average, these 20 cases had *M* = 3.20 victims, *SD* = 1.91, varying from 2 to 9 victims). For *n* = 23 victims, only the first form was filled in, and therefore it was unclear whether teachers ever had a discussion with the bullies (which actually is the most important part of the targeted intervention). After erasing these cases, the data included 249 incidents, consisting of 291 victims from *n* = 20 schools.

Importantly, some victims were enrolled in an intervention multiple times, and therefore, were double (or even triple) cases in the dataset. Specifically, in 2020-2021, seven out of the 160 victims were enrolled in a targeted intervention twice and one victim was enrolled in a targeted intervention three times. In 2021-2022, one out of the 131 victims was involved in an intervention two times, and one victim was involved in an intervention three times. Moreover, five victims were enrolled in a targeted intervention not only in 2020-2021, but also in the academic year of 2021-2022. The repeated victims were from different schools (only two repeated victims were from the same school), but it was remarkable that the bullies of the nine repeated victims *within* a school year were mostly enrolled in an intervention using the Condemning Approach (*n* = 6) and Empathy-raising approach (*n* = 3). Bullies of the five repeated victims across the two academic years were also mostly enrolled in an intervention using the Condemning approach (*n* = 4), and one was from a school implementing the Empathy-raising approach. Thus, in general, victims whose bullies received a Condemning targeted intervention were most likely to become a target of bullying again later in the school year. This could of course be either by the same or by (some) other bullies, but it was beyond the scope of this paper to systematically examine this. To prevent bias of interdependence in imputing the missing data, for the repeated victims, it was decided to only keep the information about the first targeted intervention. This resulted in a file consisting *n* = 274 unique victims (*n* = 151 year 2020-2021, *n* = 123 year 2021-2022) who were nested in 235 incidents.

**Supplementary Material 2 – Combining Targeted Intervention Data and Survey Data**

It was necessary to combine targeted intervention data and survey data to evaluate the longer-term effectiveness of targeted interventions. Moreover, because there was more missing data than anticipated, combining these datasets enabled to more reliably impute missing data based on auxiliary variables in survey data. Thus, combining targeted intervention data with survey data was a very important first step to take. A combined file was created for each academic school year separately. These two files contained students who 1) indicated to be victimized on the survey at T1 or T2, or 2) were enrolled in a targeted intervention, or 3) both. For *n* = 147 students it was possible to combine targeted intervention data with survey data based on a “research-ID”. This ID was generated for each student who had consent to participate in the Challenge project. Only researchers could attach this research-id to the student ID for the survey data collection. When school personnel started up a targeted intervention, they were asked to report the ID-number in the app (they could retrieve this ID from a password-protected student list provided by the researchers). Unfortunately, for many victims whose bullies were enrolled in a targeted intervention (*n* = 127), is was not possible to not match their targeted intervention data with their responses on the survey based on this research ID. There were various reasons for this: 1) the victims had not indicated on survey data that they had been victimized on either T1 or T2 of the two academic years or were absent during data-collection; 2) the victims included in the targeted intervention had no consent to participate in the survey data-collection of Challenge project and therefore did not have a research-ID, 3) school personnel did not (correctly) report the research-ID, 4) students received an intervention *after* the final wave of the survey (*n* = 3 in year 1 and *n* = 1 in year 2).

In year 1, for 77 out of the 151 victims that were enrolled in a targeted intervention between T1 and T3, it was possible to combine targeted intervention data with ‘victim survey data’ (i.e., the survey data from self-reported victims at T1 and T2). In year 2, for 50 out of 123 victims enrolled in a targeted intervention between T1 and T3, targeted intervention data could be combined with ‘victim survey data’. Therefore, for 127 out of the 274 victims who had bullies included in an intervention between T1 and T3, 1) students had consent *and* reported on the survey at least of T1 or T2 that they had been victimized, and 2) there was both targeted intervention data *and* some survey data.

For 20 additional students across academic years, there was no survey data because they had been absent at both T1 and T2, even though they did have consent to participate to the survey. It was therefore decided to only use them as cases in analyses on the longer-term effectiveness of victimization if imputed survey values suggested that they were victimized at T1 or T2. This implies that each imputed file could yield a distinct number of intervention students.

**Supplementary Material 3 – Multiple Imputation for Missing Data**

There was more missing data than anticipated. This missing data stemmed from various sources. First, only *n* = 198 victimized students (out of the initial 274 victimized students, thus 72.3%) were present during the follow up meeting. Only for these cases, school personnel had reported on whether victims thought that the bullying had ceased, decreased, remained the same, or had increased. Given that the sample was not large to begin with (in particular the group of students whose bullies were in the combined approach, *n* = 44), it was decided that - in contrast to the preregistration - it was necessary to impute the data to gain power. Another source of missingness was that students did not participate in all survey waves. The percentage of missingness on victimization items at W3 varied from 50.2 to 50.5% across items in academic year 2020-2021 and 52.9 to 53.3% in academic year 2021-2022. Because students’ survey victimization scores at T1 and T2 determined whether they were included in the dataset, only 3.0% had complete missingness on these victimization items. This missingness was caused by students from targeted interventions who were either absent during survey data collection or who did not have consent to participate in the survey data collection. Still, a quite large percentage of students also had missingness on the specific victimization items (about 35%), because these were not administered among younger 1-3 grade students (they only answered the item about general victimization).

It was decided to impute data of all students in the files for both academic years separately using Multiple Imputation in the mice package (version 3.16.0; van Buuren et al., 2011), implemented in R (Version 4.2.2). Various auxiliary variables were considered to improve the accurateness of missing imputation. A full matrix of auxiliary variables, predictors, and outcomes is available upon request. For interaction effects, passive imputation was applied (i.e., interactions are not imputed but computed from imputed main variables, but interactions are used to impute other variables, not including the variables that make the interaction). Below it is described how all auxiliary variables were assessed. Some of these were assessed at three waves – then time constraints were implemented: same variables were imputed between waves, and different variables were only imputed within waves.

**Auxiliary Variables from the Targeted Intervention Dataset**

**Types of victimization.** In total, 8 dummy variables were included assessing the types of victimization that students had been subjected to, including that bullies took and messed up their stuff, harassed them verbally, physically, or online, spread negative messages about them, threatened them, excluded them, spread rumours about them, or other types of bullying. These items were created based on descriptions that teachers provided, and they were coded with either 1 (victim exposed to that type of victimization) or 0 (victim not exposed to that type of victimization).

**Auxiliary Variables from the Survey Dataset.**

**Peer-nominated victimization.** Peer-nominated victimization was assessed using the proportion scores of students’ incoming nominations for three peer-nominated items (S/He is pushed and hit, S/He is called with nasty names or made fun of, S/He is usually talked about with bad tone) at the classroom level (cf. Laninga-Wijnen et al., 2024). Students could nominate an unlimited number of (cross- and same-sex) classmates and could also choose the option ‘nobody’ or skip the question. Items were internally consistent, with Cronbach’s alphas ranging from α = .73 to α = .81.

**Peer-nominated popularity.** Peer-nominated popularity was assessed using the proportion of incoming nominations on the question ‘who are the most popular children in your class?” (cf. Marks et al., 2011). For this item, students could also nominate an unlimited number of (cross- and same-sex) classmates and could also choose the option ‘nobody’ or skip the question.

**Peer-nominated likeability.** Peer-nominated likeability was assessed using the proportion of incoming nominations on the question ‘who do you like the most?” (cf. Marks et al., 2011). For this item, students could also nominate an unlimited number of (cross- and same-sex) classmates and could also choose the option ‘nobody’ or skip the question.

**Depressive symptoms at T1, T2, and T3.** Ten items from the Children’s Depression Inventory (CDI, Kovacs, 1992) were administered to assess students’ depressive symptoms. In each item, students were asked to choose one out of three statements that described how they had felt in the past two weeks, with 1 = “I am sad once in a while”, 2 = “I am sad many times”, and 3 = “I am sad all the time”. Five items were recoded so that higher scores reflect higher depression, and the ten items were averaged. Previous work has demonstrated the validity of this scale in comparable age groups (Jelinek et al., 2021). The scale was internally consistent across waves, for both academic year 2020 - 2021 and academic year 2021 - 2022, with Cronbach’s alphas ranging from α = .86 to α = .88.

**Self-esteem at T1, T2, and T3.** Self-esteem was measured using 10 items about general self-concept, derived from the Rosenberg self-esteem scale (Rosenberg, 1965). Participants responded on a 4-point Likert-scale (1 = strongly disagree, 4 = strongly agree) to items including “I feel that I have a number of good qualities” and “I feel that I am a person of worth. I am as good as anybody else”. Five negatively phrased items were recoded so that higher scores reflect higher self-esteem. Validity of this scale was found to be appropriate in previous work with comparable samples in terms of age (e.g., Donnellan et al., 2016). Therefore, items were averaged. The scale was internally consistent across waves, for both academic year 2020 - 2021 and academic year 2021 - 2022, with Cronbach’s alphas ranging from α = .88 to α = .91.

**Anxiety at T1, T2, and T3.** The SCAS-C-8 scale (Reardon et al., 2018) has been administered to assess anxiety, with items such as: “I worry sometimes that bad things will happen to me”, or “I worry about what others may think of me”. Answers were provided on a 4-point Likert scale, ranging from 1 = never to 4 = always. Previous work has shown the validity of this scale in comparable age groups (Spence et al., 2018). Items were internally consistent across waves, for both academic year 2020 - 2021 and academic year 2021 - 2022, with Cronbach’s alphas ranging from α = .83 to α = .85. Therefore, items were averaged to create a scale for anxiety in both datafiles.

**Internal causal attributions at T1 and T2.** Internal causal attributions for victimization were assessed with a scale from the ‘*Why Kids Pick on Me*’ measure, found to be valid in previous work (Visconti et al., 2013). Four items were administerd to assess the extent to which students would blame themselves, if someone might pick on them. These reasons could include being different (e.g., “because I am different from them”) and not being cool (e.g., “I do not wear cool clothes”). Responses were given on a 4-point likert scale (1 = never the reason, 4 = always the reason). The scale was internally consistent across waves, for both academic year 2020 - 2021 and academic year 2021 - 2022, with Cronbach’s alphas ranging from α = .77 to α = .89.

**References:**

van Buuren, S., & Groothuis-Oudshoorn, K. (2011). mice: Multivariate Imputation by Chained Equations

in R. *Journal of Statistical Software*, *45*(3), 1–67. <https://doi.org/10.18637/jss.v045.i03>

Cillessen, A. H., & Marks, P. E. (2011). Conceptualizing and measuring popularity. *Popularity in the peer system*, 25-56.

Donnellan, M. B., Trzesniewski, K. H., Robins, R. W., Moffitt, T. E., & Caspi, A. (2005). Low self-esteem is related to aggression, antisocial behavior, and delinquency. *Psychological science*, *16*(4), 328-335.

Jelínek, M., Květon, P., Burešová, I., & Klimusová, H. (2021). Measuring depression in adolescence: Evaluation of a hierarchical factor model of the Children’s Depression Inventory and measurement invariance across boys and girls. *Plos one*, *16*(4), e0249943.

Kovacs, M. (1992). Children's Depression Inventory manual/Multi-Health Systems.

Laninga-Wijnen, L., Garandeau, C. F., Malamut, S. T., & Salmivalli, C. (2024). The longitudinal role of classroom defending norms in victims’ psychological adjustment, causal attributions, and social comparisons. *Developmental Psychology*, *60*(3), 522.

Reardon, T., Spence, S. H., Hesse, J., Shakir, A., & Creswell, C. (2018). Identifying children with anxiety disorders using brief versions of the Spence Children’s Anxiety Scale for children, parents, and teachers. *Psychological Assessment*, *30*(10), 1342.

Rosenberg, M. (1965). Society and the adolescent self-image. Princeton, NJ: Princeton University Press

Visconti, K. J., Kochenderfer-Ladd, B., & Clifford, C. A. (2013). Children’s attributions for peer victimization: A social comparison approach. Journal of Applied Developmental Psychology, 34(6), 277–287. [https://doi.org/10 .1016/j.appdev.2013.06.002](https://doi.org/10%20.1016/j.appdev.2013.06.002)

**Supplementary Material 4 - Propensity Score Matching to Determine Control Group to Evaluate Longer-term Effectiveness**

To compare the longer-term development of victimization among students enrolled in interventions vs. those who were not, survey data was used. A total of 4,311 students reported that they were victimized on the survey, but only a fraction of them (*n* = 274, 6.4%) were enrolled in a targeted intervention. Only 147 out of these 274 intervention victims had consent to participate in the survey (i.e., “survey-consenting intervention students”). Therefore, only for this small sample it was possible to examine the longer-term effectiveness of targeted interventions. It would be inappropriate to compare this small number of “survey-consenting intervention students” with the disproportionally high number of survey-reported victims who were *not* enrolled in an intervention. Therefore, propensity score matching was applied to obtain an equally sized control group that would be similar to intervention students in biological sex, school level, self-reported and peer-reported victimization, age, anxiety, and peer acceptance. These characteristics were expected to relate to the likelihood that students would be included in a targeted intervention or not.

A first important point to mention is that the sample of “survey-consenting intervention victims” varied across the 50 imputed datasets. This happened because for only 127 victims there was information of victimization at baseline. There were 20 other victims who were enrolled in the targeted intervention and who had consent to participate in the survey, but who had been absent on both T1 and T2 of the survey. It was decided to impute their missing values, and therefore it could vary across imputed datasets whether they could be considered as victim or not. Thus, for each dataset, there was a slightly different intervention group (it varied from *n* = 129 to *n* = 143). Then, for each dataset, propensity score matching was applied to find an equally populated control group who were similar to intervention students based on biological sex, school level, and victimization. Importantly, students who were enrolled in an intervention in year 2 were not allowed to serve as control group for year 1. First, a control group was determined for year 1. After defining the control group at year 1, these students were erased from the files of year 2, to prevent that the same students were included twice. Then, propensity score matching was applied on the file of year 2.

Propensity score matching was applied on all 50 datasets, per school year. This implies that for each dataset, resulting in a slightly different pool of matches for the control group. This however is consistent with stochastic actor modelling. To prevent bias in imputation, it is important that a level of “uncertainty” is added to the data files. This “uncertainty” is reflected by having multiple data-files, and consequently, by having slightly distinct “control groups” for each imputed file. Therefore, when referring to characteristics of the control group, this study will only refer to percentages (averaged across imputed files) and not to absolute numbers. The matching went well: Pooled adjusted standardized mean differences were all well below .10, indicating that the control group and intervention group were highly comparable in characteristics (Table S1).

| Table S1. Pooled Balance Table with Adjusted Mean Differences per Matching Characteristic | | |
| --- | --- | --- |
| Variables | Dataset 2020-2021 | Dataset 2021-2022 |
| Biological sex | .000 | -.004 |
| School level | -.005 | .005 |
| Self-reported victimization | -.024 | -.039 |
| Peer-nominated victimization | .027 | -.016 |
| Age | .018 | .030 |
| Anxiety | -.021 | -.003 |
| Peer Acceptance | .027 | -.041 |
| Distance | .002 | .009 |

**Supplementary Material 5 - Transparent Changes to Pre-registration**

The current study is based on a pre-registration and its subsequent adaptation. Initially, it was planned to conduct a two-part study: Study 1 would assess short-term effectiveness without a control group, while Study 2 would evaluate longer-term effectiveness, both in average and dyadic victimization, with a control group. Researchers of the current study initially intended to avoid imputing data due to a lack of auxiliary variables for each study. However, the significant amounts of missing data necessitated a shift in approach, as detailed in the adapted pre-registration.

The following modifications were pre-registered:

1. Instead of conducting a 1-2 study, researchers opted for a single study utilizing a comprehensive dataset that combined targeted intervention data with survey data. This larger dataset enabled the generation of auxiliary variables necessary for imputing missing data.
2. The initial plan for part 2 of the 1-2 study was to only select 4-9 grade students who indicated at T1 that they were victimized at least 2 or 3 times a month on at least one of the five specific victimization items. However, researchers discovered that this was a too strict criterium that led to an overly large amount of omitted cases. Specifically, with this criterium, researchers only would end up with *n* = 32 victims whose bullies were enrolled in an intervention and for whom researchers could analyze their self-reported victimization over time. For one of them the intervention even took place *after* the end-of-the year survey, leaving an overly small sample of *n* = 31 students to analyze. The most important reasons for this small number of cases was that: 1) many students involved in a targeted intervention had no research-ID, either because they did not participate in the surveys or because teachers did not correctly report them - this was true for almost 100 students; 2) students did not report that the victimization did take place at T1 (but they *did* indicate to be victimized at T2, and after T2 they were included in the intervention); 3) students indicated on a general item asking about victimization that they were victimized, but not in the specific ways researchers asked from them; 4) students indicated to be victimized once or twice, rather than two or three times a month, 5) students were from grades 1 - 3. To address these issues, selection criteria were broadened, to include 1st to 9th grade students who reported victimization at either T1 or T2, provided T2 occurred before the targeted intervention, regardless of the frequency or specificity of their reports.
3. Due to overly large amounts of missingness, researchers decided to refrain from analyzing questions regarding victim-bully dyads.

In addition to these adaptations of the pre-registration, researchers decided to take the following steps that deviate from the (adapted) plan:

1. The number of victims in targeted interventions was still smaller than anticipated and the control group was disproportionately large. Therefore, researchers implemented a matching procedure. In the control group, researchers randomly selected students who matched those in the intervention group based on biological sex, age, and self-reported victimization frequency at T1 or T2.
2. Based on valuable feedback of the reviewers, researchers performed a more thorough matching procedure - researchers used 7 rather than 4 variables to match intervention students with control students.
3. researchers initially wanted to control for the duration of days between the final intervention step and the end-of-the-year assessment, but it was not possible to reliably impute data of date-variables. researchers did still control for it in sensitivity analyses, and the role of duration was non-significant. The other results in the model remained the same. Findings are available upon request.
4. For the Latent Class Structural Models (LCSM), researchers did not include interactions with biological sex and age due to an insufficient number of cases.
5. Researchers initially wanted to for the nestedness of students within schools by running a multi-level model in which researchers left the between part empty, as researchers did not have between-level predictors and to use the WLSMV estimator. Yet given only few victims per classroom and the limited sample, researchers refrained from conducting multi-level analyses and researchers applied ML estimation. The ICC was very low (ICC = .0169) suggesting that there is not much variance to be explained at the school-level.
6. Researchers ran analyses in R(lavaan, semTools) rather than in Mplus, so that they were consistent in open-source software used.
